# Efficacy of 4′-[methyl-11C] thiothymidine PET/CT before and after neoadjuvant therapy for predicting therapeutic responses in patients with esophageal cancer: a pilot study

**DOI:** 10.1186/s13550-019-0478-9

**Published:** 2019-01-30

**Authors:** Masatoshi Hotta, Ryogo Minamimoto, Kazuhiko Yamada, Kyoko Nohara, Daisuke Soma, Kazuhiko Nakajima, Jun Toyohara, Kei Takase

**Affiliations:** 1 Division of Nuclear Medicine, Department of Radiology, National Center for Global Health and Medicine, 1-21-1, Toyama, Shinjuku-ku, Tokyo, 162-8655 Japan; 2 Department of Surgery, National Center for Global Health and Medicine, 1-21-1, Toyama, Shinjuku-ku, Tokyo, 162-8655 Japan; 3 Functional Brain Research Team for Neuroimaging, Tokyo Metropolitan Institute of Gerontology, 35-2, Sakae-cho, Itabashi-ku, Tokyo, 173-0015 Japan; 4 Department of Diagnostic Radiology, Tohoku University Hospital, 1-1, Seiryo-machi, Aoba-ku, Sendai, Miyagi 980-8574 Japan

**Keywords:** 4DST, Esophageal cancer, PET/CT, FDG, Therapeutic response

## Abstract

**Background:**

4′-[Methyl-11C] thiothymidine (4DST) has been introduced as a new cell proliferation imaging PET tracer that incorporates into DNA directly. The aim of this prospective study was to evaluate the efficacy of 4DST PET/CT for predicting responses to neoadjuvant therapy in patients with esophageal cancer comparing with FDG PET/CT.

**Methods:**

Twenty-six patients who had pre- and post-therapeutic 4DST and FDG PET/CT and underwent esophagectomy following neoadjuvant therapy were used for the analysis. Based on pathological findings, patients were divided into two groups: non-responders and responders. The maximum standardized uptake value (SUVmax), metabolic tumor volume, total lesion glycolysis, and total lesion proliferation of the primary lesion were measured for FDG and 4DST PET.

**Results:**

The pathological diagnosis revealed 16 responders and 10 non-responders. Non-responders showed significantly higher 4DST post-therapeutic SUVmax (_post_SUVmax) than responders, whereas FDG _post_SUVmax showed no statistically significant difference (non-responders vs. responders: 4DST, 6.7 vs. 3.3, *p* = 0.001; FDG, 6.1 vs. 4.5, *p* = 0.11). Responders showed a greater reduction in percentage changes of 4DST and FDG SUVmax (ΔSUVmax) from baseline to post-therapeutic PET (non-responders vs. responders: 4DST, − 2.9% vs. − 56.7%, *p* < 0.001; FDG, − 36.3% vs. − 72.6%, *p* < 0.001). In ROC analysis, ΔSUVmax and _post_SUVmax with 4DST provided great diagnostic performance for predicting responses (area under the curve: 4DST ΔSUVmax = 0.92, 4DST _post_SUVmax = 0.88).

**Conclusions:**

4DST PET/CT has a great potential for predicting pathologic response to neoadjuvant therapy in patients with esophageal cancer; it may be slightly superior to that with FDG PET/CT.

## Background

The prognosis of patients with advanced esophageal cancer continues to be poor, despite advances in management. Neoadjuvant chemotherapy or chemoradiation therapy before esophagectomy is a standard-of-care and commonly applied in clinical practice for locally advanced and operable esophageal cancer [1–3]. Once neoadjuvant therapy is completed, assessment of response is necessary [3]. When a persistent local lesion is indicated, esophagectomy is strongly recommended because the presence of residual tumor after neoadjuvant therapy in the resected specimen leads to shorter overall survival [3–5]. On the contrary, if there is no evidence of residual viable lesion, surveillance can be a possible option [3]. Generally, FDG PET/CT and/or contrast-enhanced chest CT is used for the evaluation of treatment response of neoadjuvant therapy in esophageal cancer [3]. It is sometimes difficult to distinguish a viable residual tumor form reactive changes with a chest CT. In contrast, FDG PET/CT provides a more accurate diagnosis compared to that with chest CT due to its evaluation of metabolic activity. However, the value of FDG-PET/CT for evaluating response to neoadjuvant therapy in esophageal cancer is still controversial [6–8], so it is basically not recommended for the selection of patients for esophagectomy following neoadjuvant therapy [3].

Recently, Toyohara et al. developed 4′-[methyl-11C] thiothymidine (4DST) as a new DNA synthesis imaging agent [9, 10]. Although 3′-fluoro-3′-deoxythymidine (FLT) has been established as a cell proliferation PET tracer, 4DST has advantages for proliferation measurement [11]. 4DST incorporates into DNA directly, whereas FLT does not incorporate into DNA and reflects salvage pathway of DNA synthesis [12]. We have previously reported on the great potential of 4DST PET/CT for proliferation imaging in malignancies such as lung cancer and renal cell carcinoma [13–15]. In addition, Hoshikawa et al. have reported that 4DST PET shows a higher prognostic value in patients with head and neck carcinoma compared to FDG PET [16].

These results suggest that 4DST PET can potentially predict a response to neoadjuvant therapy in esophageal cancer. The aim of this study was to evaluate the diagnostic value of 4DST for predicting response to neoadjuvant therapy in patients with esophageal cancer as compared to that with FDG.

## Methods

### Patients

**Fig. 1:**
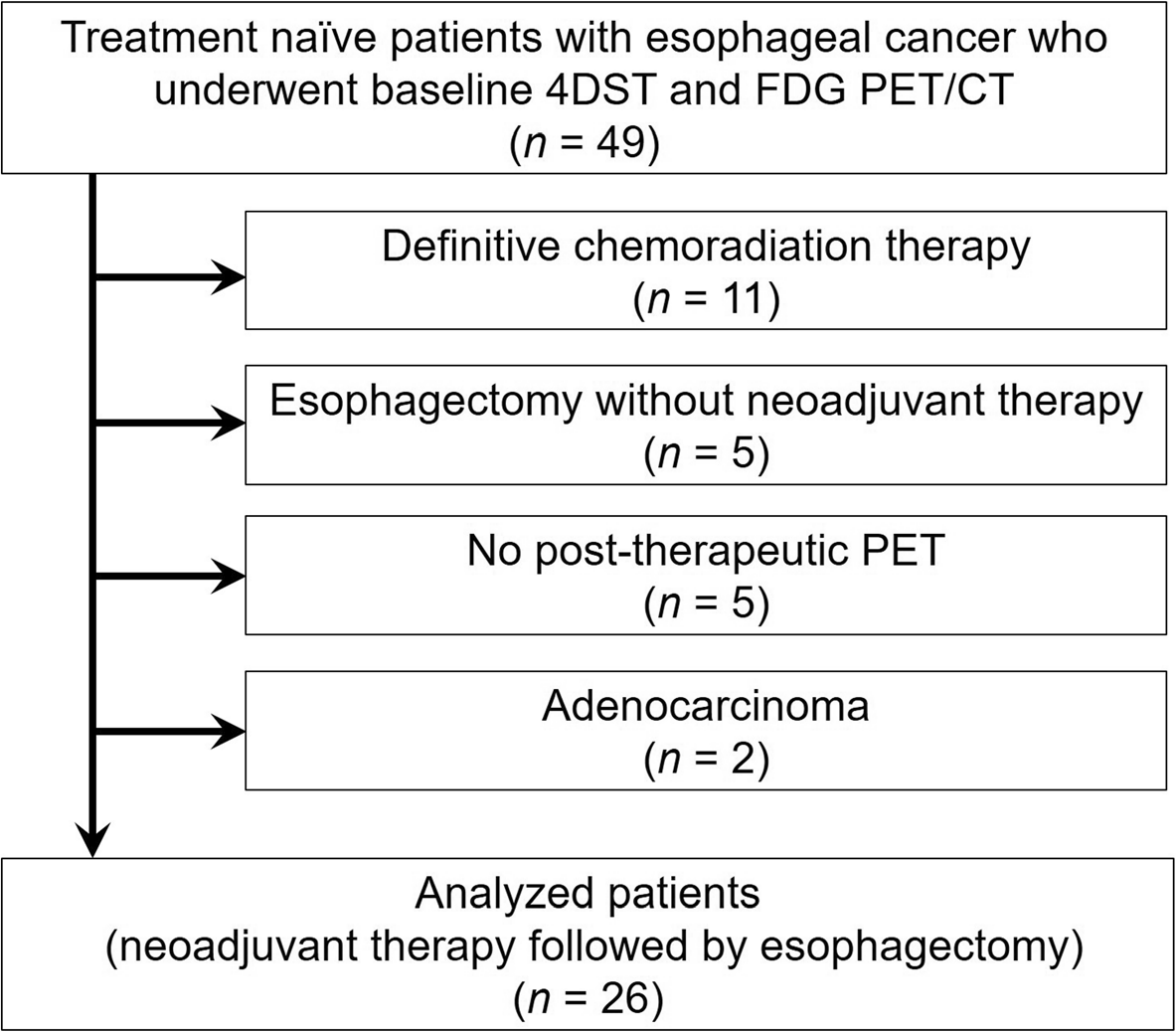
Flow diagram of patient selection

This prospective study was approved by the institutional review board of our hospital, and written informed consent was obtained from all patients. We enrolled patients with biopsy-proven esophageal cancer. A total of 49 consecutive treatment-naïve patients underwent baseline 4DST and FDG PET/CT from August 2015 to September 2018 and were assessed for eligibility for this prospective study (Fig. 1). Among them, 11 patients were treated with definitive chemoradiation therapy (8 patients declined to undertake esophagectomy, and 3 patients were regarded as inoperable due to newly diagnosed lung and/or bone metastasis), 5 patients were treated with esophagectomy without neoadjuvant therapy, and 5 patients did not have 4DST and/or FDG PET/CT after neoadjuvant therapy. In addition, 2 patients with adenocarcinoma were excluded because of the different biological entity between adenocarcinoma and squamous cell carcinoma. Therefore, we analyzed 26 patients with esophageal squamous cell carcinoma (23 men and 3 women, mean ± SD age 66.4 ± 9.7 years). They all underwent esophagectomy following neoadjuvant therapy. Exclusion criteria for these 26 patients were (1) uncontrolled diabetes or (2) non-avid tumors on FDG or 4DST PET/CT, but no patients met these exclusion criteria. Patients’ demographics including tumor markers are shown in Table 1. Regarding the regimen of neoadjuvant therapy, the chemotherapy consisted of fluoropyrimidine with platinum for all patients. The total radiation dose for the radiation therapy was 40.0 Gy (*n* = 11/12 [91.7%]) or 60 Gy (*n* = 1/12 [8.3%]) delivered in daily fractions.

**Table 1:** Patients demographics

Demographics	Number
Patients	26
Sex, male	23
Mean age years (standard deviation)	66.4 (9.7)
Location	
Upper	7
Middle	14
Lower	5
Clinical T-stage	
cT2	2
cT3	13
cT4	11
Clinical N-stage	
cN0	6
cN1	6
cN2	13
cN3	1
Neoadjuvant therapy	
Chemotherapy	14
Chemoradiotherapy	12
Tumor marker	
SCC (ng/mL) [median, (range)]	2.2 (0.6, 8.1)
CEA (ng/mL) [median, (range)]	2.2 (0.2, 8.6)

### 4DST-PET/CT examination

The 4DST was synthesized using a previously described method [17]. All subjects fasted for 5 h before receiving the intravenous injection of 4DST with a mean of 361 ± 46 MBq (pre-treatment) and 361 ± 22 MBq (post-treatment). PET/CT images were obtained from skull base to pelvis 40 min after intravenous injection and performed using either of cross-calibrated three PET/CT systems ((1) Biograph mCT S20: Siemens Medical Solutions, Erlangen, Germany; (2) Biograph 16: Siemens Medical Solutions; and (3) Discovery PET/CT 600: GE Healthcare, Pewaukee, WI, USA). These systems equipped a PET scanner and a multi-detector-row CT scanner (16 detectors). Low-dose CT with shallow breathing was firstly performed initially and used for both attenuation correction and also image fusion. Low-dose CT data for the Biograph mCT S20 was acquired at 120 kVp using an auto-exposure-control system, and a beam pitch of 0.8, slice thickness of 5 mm. That data for the Biograph 16 was acquired at 120 kVp using an auto-exposure-control system, beam pitch of 0.833, and a slice thickness of 5 mm. Finally, that data for the Discovery PET/CT 600 was acquired at 120 kVp using an auto-exposure-control system, beam pitch of 0.938, and a slice thickness of 3.75 mm. Emission images were acquired in three-dimensional mode (Biograph mCT S20: 3.0 min per bed position; Biograph 16: 2.5 min per bed position; and Discovery PET/CT 600: 2.5 min per bed position). PET data were reconstructed with an ordered subset expectation maximization (OSEM) algorithm. It employed 3 iterations and 16 subsets for the Discovery PET/CT 600; 3 iterations, 8 subsets for the Biograph 16, and 2 iterations; and 21 subsets combined with time of fright for the Biograph mCT S20. A Gaussian filter was used for post-smoothing filter in all cases. It had a full-width at half-maximum of 5 mm. For each patient, the same scanner was used for pre- and post-therapeutic PET/CT.

### FDG-PET/CT examination

An in-house cyclotron and automated synthesis system (F200; Sumitomo Heavy Industries, Shinagawa, Tokyo, Japan) was used on the basis of the authorized procedure to synthesize FDG. Patients were instructed to fast for at least 5 h before intravenous injection of FDG, fixed at 5.0 MBq/kg. PET/CT images were acquired 60 min after injection. They were acquired with the same PET/CT scanner and manner as were the 4DST PET/CT images.

### Image analysis

All 4DST and FDG PET/CT images were analyzed by two board-certified nuclear medicine physicians who were unaware of the clinical data. The primary tumor was defined as the volume of interest (VOI) and delineated on the 4DST and FDG PET scans using semi-automatic gradient-based delineation software (MIM Software, Cleveland [OH], USA). Its superiority over manual and threshold methods has been validated [18]. The following PET quantitative values were evaluated for the primary tumor: maximum standardized uptake value (SUVmax), metabolic tumor volume (MTV), total lesion glycolysis (TLG), and total lesion proliferation (TLP). The MTV was calculated by summing up the areas within each two-dimensional transverse tumor contour multiplied by the corresponding slice thickness automatically by the software. The TLG was calculated by multiplying MTV by SUVmean in the FDG study and the TLP in the same way for the 4DST study. Each parameter was assessed both pre-therapy (_pre_SUVmax, _pre_MTV, _pre_TLG, and _pre_TLP) and post-therapy (_post_SUVmax, _post_MTV, _post_TLG, and _post_TLP), and then percent changes calculated [for SUVmax (ΔSUVmax) for MTV (ΔMTV), for TLG (ΔTLG), and for TLP (ΔTLP). With FDG PET, tumor response was also evaluated using PET Response Criteria in Solid Tumors (PERCIST) version 1.0. [19]. The lean body mass-corrected SUV peak was measured by MIM Software.

### Histopathologic assessment

The reference standard for the diagnosis of response to neoadjuvant therapy was histopathologic examination of the resected primary tumor specimen, which was performed by an experienced pathologist based on hematoxylin and eosin-stained tissue sections. The degree of pathologic response to neoadjuvant treatment was graded as follows [20]: (grade 0, ineffective) no recognizable histological therapeutic effect, (grade 1, slightly effective) apparently viable cancer cells account for 1/3 or more of the tumor tissue, but there is some evidence of degeneration of the cancer tissue or cells grade 1a: viable cancer cells account for 2/3 or more tumor tissue. Grade 1b: viable cancer cells account for 1/3 or more, but < 2/3, of tumor tissue, (grade 2; moderately effective) viable cancer cells account for less than 1/3 of tumor tissue, (grade 3; markedly effective) no viable cancer cells. Patients who showed a grade 0–1a pathologic response were considered non-responders, while patients who were classified as grade 1b–3 were considered responders [21, 22].

### Statistical analysis

No power analysis was performed because of the lack of previous studies on the topic. Data are expressed as mean ± SD. The association between clinical baseline characteristics and responders versus non-responders was studied using Student’s *T* test for parametric continuous parameters, Mann-Whitney *U* test for non-parametric continuous parameters, and Fisher’s exact test for categorical parameters. Mann-Whitney *U* test was used to compare quantitative values of 4DST and FDG PET parameters between the groups (responder and non-responder). Receiver operating curve (ROC) analyses (providing area-under-the-curve (AUC) values) were performed to evaluate the diagnostic ability of the FDG and 4DST PET parameters to distinguish responders from non-responders. Statistical significance was considered to be present for values of p less than 0.05.

## Results

### Clinical data

The pathological diagnosis revealed 10 non-responders (grade 0: 1, grade 1a: 9) and 16 responders (grade 1b: 4, grade 2: 9, and grade 3: 3). The clinical characteristics of responders and non-responders are described in Table 2. None of the baseline characteristics, including tumor markers, were related to the pathologic response to neoadjuvant therapy. The mean interval between pre-treatment FDG PET and pre-treatment 4DST PET and between post-treatment FDG PET and post-treatment 4DST PET was 4.7 ± 5.2 and 5.7 ± 4.4 days, respectively. The mean interval between esophagectomy and post-treatment FDG PET and between esophagectomy and post-treatment 4DST PET was 14.4 ± 6.7 and 9.0 ± 7.0 days, respectively. Thirteen patients underwent PET/CT with Discovery PET/CT 600, 6 patients with Biograph 16 and 7 patients with Biograph mCT S20.

**Table 2:** Clinicopathologic characteristics comparing between responders and non-responders

	Responders	Non-responders	*p* value
No. patients	16	10	
Sex, male	14	9	1.00
Mean age years (standard deviation)	65.2 (10.7)	71.6 (4.9)	0.072
Location			0.17
Upper	3	4	
Middle	8	6	
Lower	5	0	
Clinical T-stage			0.141
cT2	1	1	
cT3	6	7	
cT4	9	2	
Clinical N-stage			0.090
cN0	1	5	
cN1	4	2	
cN2	10	3	
cN3	1	0	
Neoadjuvant therapy			0.051
Chemotherapy	6	8	
Chemoradiation therapy	10	2	
Tumor marker			
SCC (ng/mL) [median (range)]	1.9 (0.7–6.2)	2.8 (0.6–8.1)	0.36
CEA (ng/mL) [median (range)]	2.4 (0.6–6.4)	2.1 (0.2–8.6)	0.90

### PET/CT parameters

**Fig. 2:**
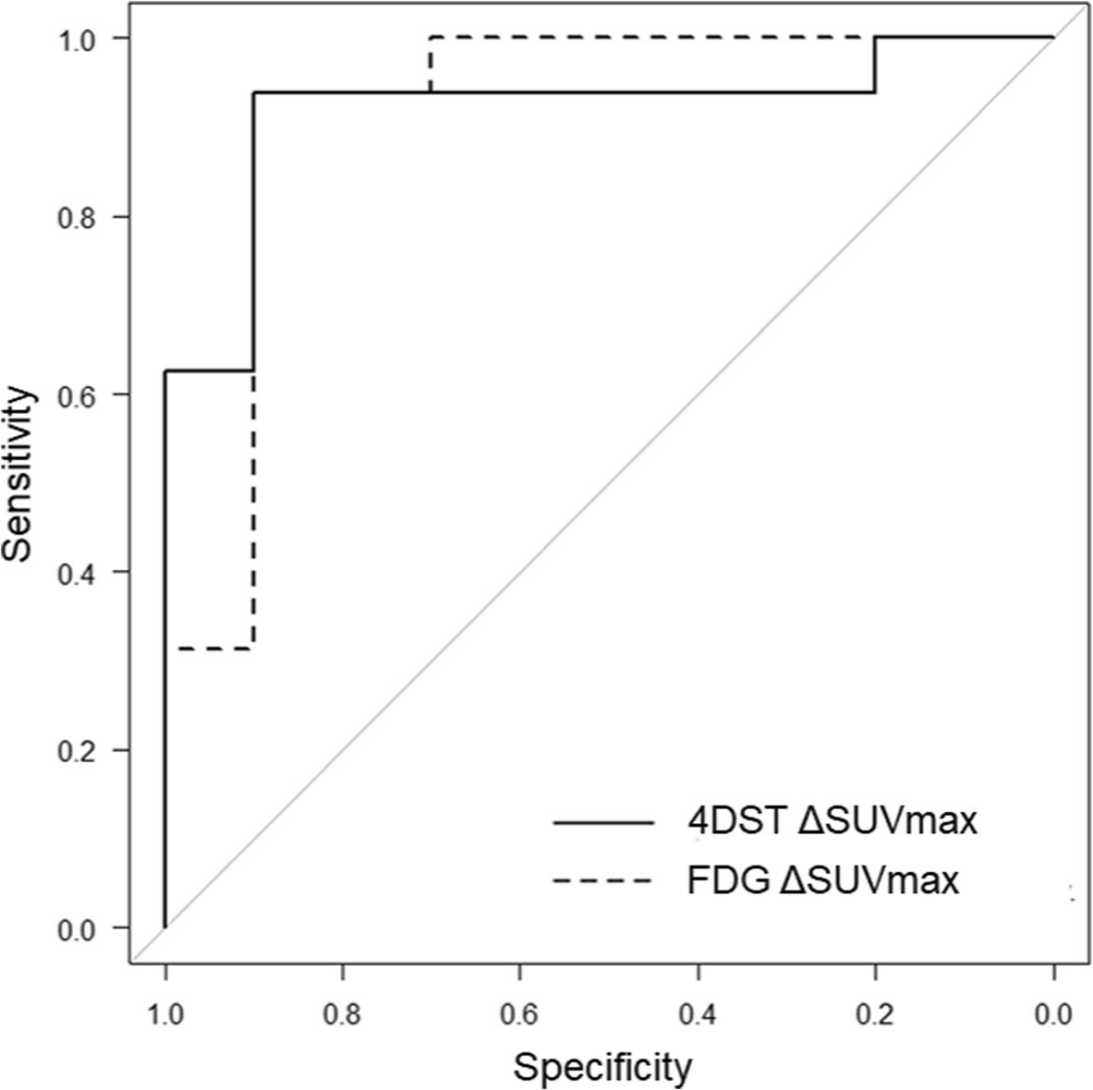
Receiver operating characteristic curve comparing between 4DST ΔSUVmax and FDG ΔSUVmax

**Fig. 3:**
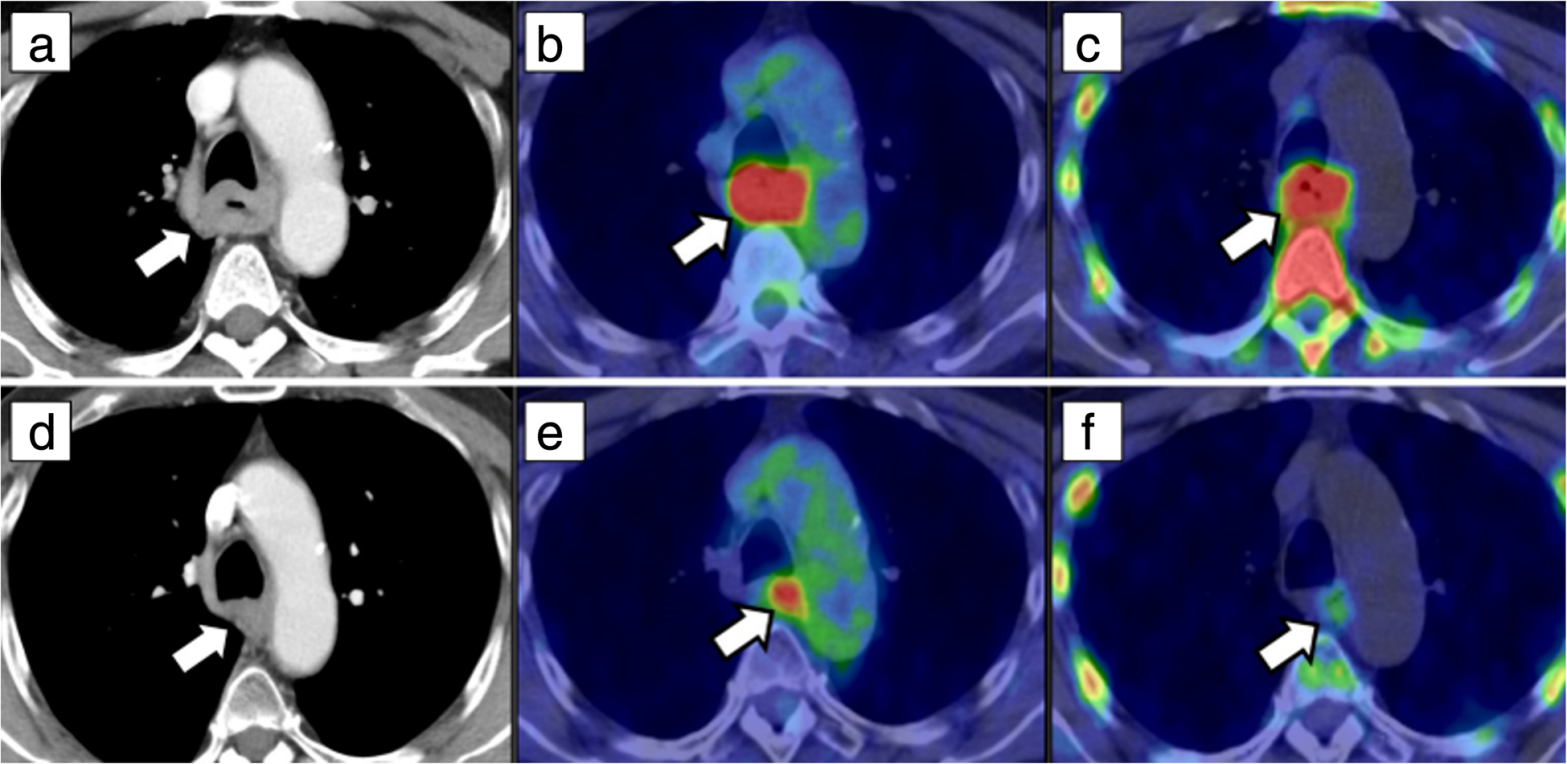
A 68-year-old man with esophageal cancer treated by neoadjuvant chemoradiation therapy followed by esophagectomy. Baseline images (CT (**a**), FDG-PET/CT (**b**), and 4DST-PET/CT (**c**)). Post-neoadjuvant therapy images (CT (**d**), FDG-PET/CT (**e**), and 4DST-PET/CT (**f**)). Baseline chest CT showed esophageal cancer (**a**: arrow) in the upper thoracic region, and FDG and 4DST PET/CT demonstrated uptake into the primary lesion (**b**, **c**: arrow). Maximum standardized uptake value (SUVmax) of FDG and 4DST was 13.5 and 6.2, respectively. After neoadjuvant chemoradiation therapy, the tumor showed reduction in size (**d**: arrow). Post-therapeutic FDG-PET/CT demonstrated FDG-avid (SUVmax = 5.3) primary lesion (**e**: arrow) suggesting the possibility of inadequate response. In contrast, 4DST-PET/CT showed relatively low uptake (SUVmax = 2.5) into the tumor (**f**: arrow) indicating this patient was a responder. Subsequently, the patient underwent esophagectomy and pathologically confirmed as responder status (grade 2)

Table 3 demonstrates the values of parameters of pre- and post-therapeutic FDG and 4DST PET, and their percent changes, comparing between non-responders and responders. In post-therapeutic PET, 4DST _post_SUVmax was statistically lower for responders than those for non-responders (*p* = 0.001). As for percent changes of PET parameters between pre- and post-neoadjuvant therapy, FDG ΔSUVmax, ΔTLG, 4DST ΔSUVmax, and ΔTLP showed statistically greater reduction in responders than in non-responders. The AUCs of these PET parameters for discrimination of responders from non-responders are shown in Table 4, and ROC curve comparing between FDG ΔSUVmax and 4DST ΔSUVmax are described in Fig. 2. The representative case is demonstrated in Fig. 3.

**Table 3:** FDG and 4DST PET parameters pre- and post-neoadjuvant therapy, comparing between responders and non-responders (median, (interquartile range))

Parameters	Responders	Non-responders	*p* value
FDG			
_pre_SUVmax	16.9 (13.1, 19.5)	10.0 (8.0, 13.9)	0.018
_post_SUVmax	4.5 (3.2, 5.4)	6.1 (4.2, 7.9)	0.11
ΔSUVmax (%)	− 72.6 (− 78.4, 67.2)	− 36.3 (− 49.9, − 18.7)	< 0.001
_pre_MTV	18.7 (7.09, 32.9)	8.7 (4.6, 25.3)	0.29
_post_MTV	3.1 (2.1, 6.9)	2.5 (1.8, 5.6)	0.75
ΔMTV (%)	− 70.4 (− 86.1, − 60.6)	− 59.1 (− 76.2, − 45.7)	0.27
_pre_TLG	150.5 (70.8, 249.8)	38.3 (19.6, 160.8)	0.14
_post_TLG	8.7 (5.4, 23.7)	12.8 (6.3, 23.5)	0.60
ΔTLG (%)	− 90.2 (− 95.1, − 86.2)	− 65.3 (− 82.7, − 58.2)	0.020
4DST			
_pre_SUVmax	9.2 (5.9, 10.3)	6.8 (4.8, 8.5)	0.21
_post_SUVmax	3.3 (2.9, 5.2)	6.7 (5.7, 7.8)	0.001
ΔSUVmax (%)	− 56.7 (− 65.6, − 40.8)	− 2.9 (− 10.5, 15.9)	< 0.001
_pre_MTV	17.8 (9.7, 36.9)	15.1 (3.4, 34.9)	0.53
_post_MTV	2.1 (1.4, 3.6)	4.0 (1.6, 10.5)	0.43
ΔMTV (%)	− 86.9 (− 92.0, − 72.9)	− 54.3 (− 75.1, − 25.1)	0.11
_pre_TLP	66.9 (37.1, 201.9)	48.8 (15.5, 88.0)	0.34
_post_TLP	5.3 (2.9, 10.9)	11.9 (6.5, 34.1)	0.102
ΔTLP (%)	− 91.6 (− 95.1, − 87.0)	− 54.4 (− 75.6, − 2.3)	0.020

*SUVmax* maximum standardized uptake value, *MTV* metabolic tumor volume, *TLG* total lesion glycolysis, *TLP* total lesion proliferation

**Table 4:** ROC analysis for discriminating responders from non-responders using FDG and 4DST PET parameters

Parameters	AUC (95% CI)	Optimal cutoff value	Sensitivity	Specificity	Accuracy	PPV	NPV
FDG							
_pre_SUVmax	0.78 (0.58–0.98)	≥ 10.7	0.600	0.938	0.808	0.857	0.789
ΔSUVmax (%)	0.92 (0.78–1.00)	≤ − 60.3	0.900	0.875	0.885	0.818	0.933
ΔTLG (%)	0.78 (0.56–0.99)	≤ − 84.7	0.800	0.812	0.808	0.727	0.867
4DST							
_post_SUVmax	0.88 (0.75–1.00)	≤ 4.00	1.000	0.625	0.769	0.625	1.000
ΔSUVmax (%)	0.92 (0.80–1.00)	≤ − 19.5	0.900	0.938	0.923	0.900	0.938
ΔTLP (%)	0.78 (0.57–0.99)	≤ − 86.7	0.800	0.812	0.808	0.727	0.867

*SUVmax* maximum standardized uptake value, *MTV* metabolic tumor volume, *TLG* total lesion glycolysis, *TLP* total lesion proliferation, *PPV* positive predictive value, *NPV* negative predictive value

PERCIST 1.0 criteria provided diagnostic ability for discriminating responders with sensitivity of 1.000, specificity of 0.727, accuracy of 0.769, positive predictive value (PPV) of 0.400, and negative predictive value (NPV) of 1.000, when patients with partial metabolic response (PMR) and complete metabolic response (CMR) are defined as patients with response, and patients with stable metabolic disease (SMD) and progressive metabolic disease (PMD) are considered as patients without response.

## Discussion

The purpose of this study was to investigate whether 4DST-PET is useful for predicting the treatment response in patients with esophageal cancer, compared to the usefulness of FDG-PET. 4DST ΔSUVmax provided high AUC values to distinguish treatment responders among all PET parameters. In addition, 4DST _post_SUVmax was also helpful for predicting response.

While FDG _post_SUVmax was not statistically different between responders and non-responders, 4DST _post_SUVmax were statistically higher for responders and showed great diagnostic performance in the discrimination of responders (AUC = 0.88). It has been reported that post-therapeutic FDG-PET is useful for evaluation of tumor response in esophageal cancer [23–25], which is concordant with our results. A possible explanation why 4DST _post_SUVmax showed greater diagnostic value than that of FDG is that FDG accumulation can be affected by inflammation due to chemotherapy or chemoradiation therapy [26, 27], whereas the influence of these therapies for 4DST may not be as significant. Indeed, high tumor selectivity of 4DST, which enables discrimination between tumor and inflammation, has been demonstrated in the rodent model [28]. The other possible reason is that 4DST simply measures tumor proliferation more accurately than FDG does. 4DST uptake corresponded well to Ki-67 [29], and in lung cancer, Minamimoto et al. have reported 4DST shows a better correlation with Ki-67 than FDG does [13]. Therefore, 4DST may have the potential to reflect tumor viability more precisely than FDG, not only in lung cancer but also in esophageal cancer. The higher accumulation of 4DST is likely to indicate more residual viable cancer cells.

Both FDG ΔSUVmax and 4DST ΔSUVmax were useful for evaluating response, and PERCIST 1.0 also provided good diagnostic performance, whereas 4DST ΔSUVmax showed higher accuracy than PRECIST 1.0. It has been reported that percent changes of FDG uptake (including PERCIST 1.0) between pre- and post-treatment are effective for the evaluation of response in esophageal cancer [21, 23, 30]. In PERCIST 1.0, the cutoff value for the response is set at more than or equal to 30%. However, several studies suggested optimal cutoff values for response evaluation in esophageal cancer as high as 50–60% [21, 23, 31]. This percentage was similar to optimal cutoff values of FDG ΔSUVmax in our study (60.3%), which showed higher diagnostic performance than when using 30% as a cutoff value. Indeed, it is frequently difficult to determine the optimal cutoff value in esophageal cancer, because non-responders also tend to show reduction of FDG accumulation [22, 23, 30]. In fact, the reduction rate of FDG ΔSUVmax in non-responder was not small (median FDG ΔSUVmax = − 36.3%) in this study as well. Conversely, in 4DST PET, non-responders showed almost no change from baseline PET, whereas responders showed a great reduction of uptake (median 4DST ΔSUVmax: non-responder = − 2.9%, responder = − 56.7%). These results indicate that persistent 4DST uptake into primary lesion after treatment is highly suggestive of inadequate response. As such, the percent changes of 4DST accumulation are likely to be an easy-to-use marker in clinical practice.

FDG _pre_SUVmax was higher for responders than for non-responders, which was statistically significant (*p* = 0.018). Although various studies have failed to demonstrate the prognostic value of the baseline SUVmax in FDG-PET [26, 32–34], some reported higher FDG SUVmax in initial PET correlated with a higher rate of complete histological response [35], and even with better disease-free survival [36]. Some suggested this is because higher FDG SUVmax is related to higher proliferation cells that are rapidly proliferating may better respond to chemotherapy or chemoradiation therapy [37]. However, if this theory is true, 4DST _pre_SUVmax of responders should have been statistically higher than that of non-responders. This point should be discussed in future studies.

Further, based on ΔSUVmax, the ability for the differentiation of responders from non-responders were almost identical as depicted in ROC (Fig. 2); however, a few cases showed discrepancy between FDG and 4DST ΔSUVmax (*n* = 2). As discussed earlier, a possible explanation for this discrepancy could be that FDG accumulation can be affected by inflammation due to chemotherapy or radiation therapy, whereas the influence of these therapies for 4DST may not be significant. This issue needs to be discussed in further studies with large sample size.

This is the first study to report the utility of 4DST for the evaluation of therapeutic response in malignancy. Gerbaudo et al. has reported the efficacy of FLT, of which biodistribution is like 4DST [17], for the evaluation of treatment response in esophageal cancer [38]. Although the number of patients was limited in their study, the authors first showed that in patients with esophageal adenocarcinomas, FLT-PET demonstrated early therapy-induced decrease in tumor proliferation in response to treatment. The difference in the reduction in tumor FLT uptake during treatment between responders and non-responders was statistically significant, but for FDG, it was not. This was probably due to the fact that FLT uptake was not affected as much as FDG was by radiation induced inflammation. On the other hand, the difference in the reduction in tumor FLT uptake at after completion of therapy between responders and non-responders was no longer significant. Gerbaudo and colleagues explained that at the end of chemoradiation treatment, FLT uptake could have been affected by the continuous effect of radiation, which diminished the proliferative capacity of remaining viable cells. In contrast, _post_SUVmax and ΔSUVmax in 4DST were useful to distinguish responders from non-responders at the end of therapy in our study. The advantage of 4DST over FLT in proliferation measurement is presented in vivo analysis [10]. Namely, FLT is not incorporated into DNA and reflects salvage pathway of DNA synthesis [12], whereas 4DST incorporates into DNA directly [9]. This feature may be one of the possible reasons to explain the discrepancy of usefulness between 4DST and FLT-PET in the assessment of treatment response. The other reason might be that more than half of the patients in our study were not treated with radiation. Considering Gerbaudo et al. findings described above about the use of interim FLT-PET for early therapeutic monitoring in esophageal cancer, it is probable that interim 4DST-PET will also have great diagnostic potential in this setting and should be examined in future studies.

There are some limitations in this study. Firstly, it was performed in a single center with a relatively small number of patients. Larger prospective multicenter trials are necessary in the future. Secondly, the mixed population of patients in terms of neoadjuvant therapy (chemotherapy vs. chemoradiation therapy) is another limitation, but this factor was not statistically significant between responders and non-responders. Thirdly, three PET scanners were used in this study, which potentially influenced the values of the PET parameters. However, cross-calibration between the three scanners was performed, and the same scanner was used in each patient not only for pre- and post-neoadjuvant therapy scan but also for 4DST and FDG PET scan. Thus, the influence of the difference of scanners is considered minimal. Finally, a cyclotron is necessary for the production of 4DST, which may be a drawback for this C11-labeled PET tracer.

## Conclusions

ΔSUVmax and _post_SUVmax in 4DST PET can provide great diagnostic value for discriminating responders from non-responders in patients with esophageal cancer. A persistent 4DST uptake after neoadjuvant therapy strongly suggests the presence of residual viable tumor cells. 4DST PET showed a great potential for evaluation of treatment response in esophageal cancer, of which assessment is higher than that of FDG PET. This study was the first report to represent the usefulness of 4DST in evaluating therapeutic response and will perhaps stimulate future research that investigates the utility of 4DST in malignancies other than esophageal cancer.

## Abbreviations

4DST: 4′-[Methyl-11C] thiothymidine
AUC: Area-under-the-curve
CMR: Complete metabolic response
FLT: 3′-Fluoro-3′-deoxythymidine
MTV: Metabolic tumor volume
NPV: Negative predictive value
OSEM: Ordered-subset expectation maximization
PERCIST: PET Response Criteria in Solid Tumors
PMD: Progressive metabolic disease
PMR: Partial metabolic response
PPV: Positive predictive value
ROC: Receiver operating curve
SUV: Standardized uptake value
TLG: Total lesion glycolysis
TLP: Total lesion proliferation
VOI: Volume of interest
